# Machine learning modeling of cancer treatment-related cardiac events in breast cancer: utilizing dosiomics and radiomics

**DOI:** 10.3389/fonc.2025.1557382

**Published:** 2025-08-21

**Authors:** Sefika Dincer, Muge Akmansu, Oya Akyol

**Affiliations:** ^1^ Department of Radiation Oncology, Van Yuzuncu Yil University School of Medicine, Van, Türkiye; ^2^ Department of Radiation Oncology, Gazi University School of Medicine, Ankara, Türkiye

**Keywords:** dosiomics, oncologic treatment-related cardiotoxicity, machine learning, radiomics, oncology cardiology

## Abstract

**Background:**

Personalized medicine has transformed disease management by focusing on individual characteristics, driven by advancements in genome mapping and biomarker discoveries.

**Objectives:**

This study aims to develop a predictive model for the early detection of treatment-related cardiac side effects in breast cancer patients by integrating clinical data, high-sensitivity Troponin-T (hs-TropT), radiomics, and dosiomics. The ultimate goal is to identify subclinical cardiotoxicity before clinical symptoms manifest, enabling personalized surveillance strategies. It is the first study to utilize heart-segmented dosiomics in breast cancer patients.

**Methods and Materials:**

This retrospective study included clinical, dosimetric, radiomic, and dosiomic data from 42 women with localized breast cancer. Heart-specific Troponin T levels were measured 2–3 weeks post-radiotherapy, with 14 ng/L as the cutoff. Patients were grouped on this threshold to identify potential treatment-related cardiac events. Radiomics and dosiomics were extracted using PyRadiomics. Machine learning models were optimized using the Tree-based Pipeline Optimization Tool (TPOT), identifying the gradient-boosted classification as the best-performing algorithm. Feature selection was conducted using gradient-boosted recursive feature elimination. Model performance is assessed by the area under the curve (AUC).

**Results:**

A total of 111 dosiomic and 119 radiomic features were extracted per patient. The highest predictive accuracy was achieved using clinical, dosiomic, and radiomic parameters (validation cohort-AUC = 0.96), outperforming the clinical + dosimetric model (validation cohort-AUC = 0.67). Permutation tests confirmed the non-randomness of these two models results (p <0.05). Cross-validation indicated that the clinical + dosiomic + radiomic model had a fair-to-good generalizable performance (mean AUC = 80.33 ± 21%).

**Discussion:**

This study may demonstrate that radiomics and dosiomics provide superior predictive capabilities for cardiac events in breast cancer patients compared to traditional parameters.

## Introduction

Cardiovascular complications following cancer treatment represent a significant cause of mortality among cancer patients, mainly when therapies such as chemotherapy and radiotherapy induce structural and functional damage to the heart. This concern is particularly substantial for breast cancer patients, where treatments like anthracyclines, trastuzumab, pertuzumab, immune checkpoint inhibitors (ICIs), and radiotherapy can lead to cancer therapy-related cardiac dysfunction (CTRCD) (1–6). Consequently, regularly monitoring the cardiovascular status in breast cancer survivors is imperative, using diagnostic tools such as left ventricular ejection fraction (LVEF) and cardiac biomarkers like troponin and echocardiography (7–11).

Echocardiography is currently the preferred non-invasive imaging modality for diagnosing CTRCD (7, 12). Furthermore, this method often detects cardiac dysfunction only at advanced stages, leading to irreversible damage in 58% of cases (13, 14). A meta-analysis highlights that hs-cTnT measured at the 3-6th month of treatment provides superior early diagnostic value to echocardiography, emphasizing the need for its use in predicting cardiac events (15).

The European Society of Cardiology recommends evaluating serum cardiac biomarkers, such as Trop-T and NT-pro-BNP, before, during, and after treatment to detect subclinical CTRCD and tailor oncologic therapies to individual risk profiles (16). Elevated levels of these biomarkers in cancer patients without coronary artery disease are indicative of subclinical cardiac damage and are linked to reduced left ventricular function (17).

In the last decade, non-invasive imaging biomarkers have been developed to assist in personalized oncology decision support systems. Radiomics and dosiomics are just two of these methods. These techniques have shown promising results in predicting various clinical outcomes, including overall survival and recurrence in cancers such as brain, head and neck, and lung (18–22). However, the scientific field still lacks sufficient dosiomic-radiomic studies, particularly those focused on side effects rather than disease prognosis.

Accordingly, all this literature predicting treatment-related cardiac events before cancer therapy begins would be highly significant. This study aims to bridge this gap by developing a machine learning-based prediction model for early detection of treatment-related cardiac events in breast cancer patients. The model incorporates radiomics, dosiomics, and clinical parameters to identify subclinical cardiotoxicity. To our knowledge, this is the first study to utilize heart-segmented dosiomic data in adult breast cancer patients. To our knowledge, this is the first study to utilize heart-segmented dosiomic data in adult breast cancer patients, and it has the potential to serve as a precursor for future research in this area.

## Materials and methods

### Patients

**Figure 1:**
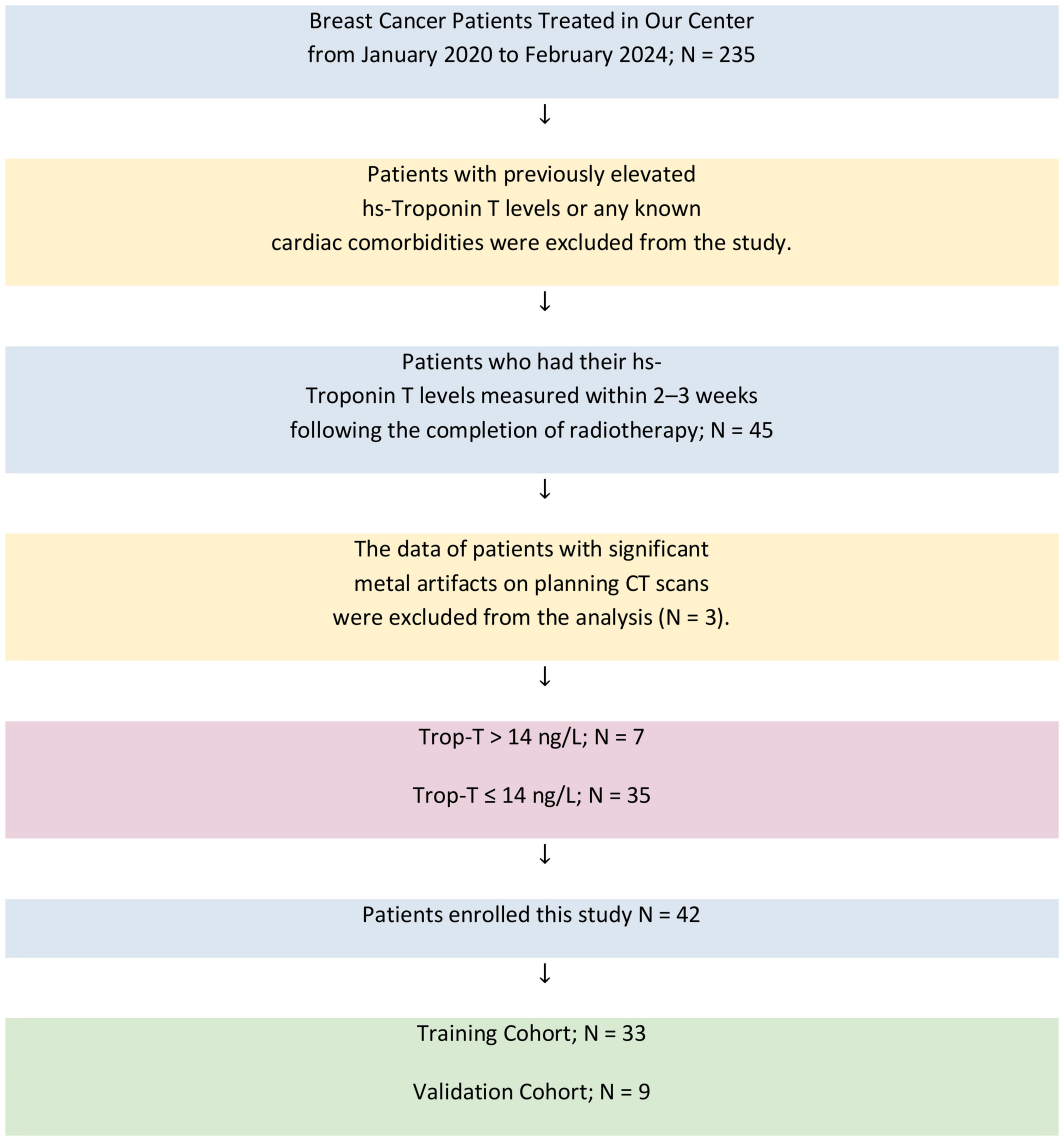
Patient selection process. N, number of patients; Trop-T, Troponin-T.

In this retrospective study, data from patients treated for localized breast cancer at our institution between January 2020 and February 2024 were analyzed. Of the 235 patients initially screened, those who had hs-TropT levels measured in the blood 2–3 weeks after the completion of radiotherapy were considered eligible. Only patients who received radiotherapy as part of their treatment were included; those treated exclusively with systemic therapies such as chemotherapy (e.g., anthracyclines, trastuzumab) were not considered. Patients with a prior history of cardiac events resulting in elevated hs-TropT levels were not eligible. Additionally, cases involving breath-controlled radiotherapy or significant metal artifacts on planning CT scans (n = 3) were omitted due to potential data quality concerns. Ultimately, 42 patients met all inclusion criteria and were retained for final analysis. As frequently recommended in the literature, the ratio between the training and validation cohort groups was set at %80/20 (33 patients in the training group and 9 patients in the validation group). The patient selection process is summarized in **Figure 1**. Clinical data included age, comorbidities, smoking status, use of cardioprotective medications, chemotherapy regimens, surgical procedures, and laterality of breast cancer. Dosimetric data included the dose of radiotherapy delivered to the heart, the presence of a boost dose, the fractionation scheme, the radiotherapy technique used, and the mean and maximum radiation doses received by the heart, as well as the heart volumes receiving 5 Gy and 25 Gy (V5-V25). Radiomic features were extracted from pre-contoured heart regions in the planning CT scans using advanced statistical methods, and dosiomic features were derived from the radiotherapy plan dose distributions through similar statistical processing. The dosiomic data were derived from the originally planned dose distribution, with the fractionation value (hypofractionation vs. normofractionation) included as a separate variable in the model. The left-right breast distinction was not defined as an independent variable but incorporated as a separate parameter in the clinical and clinical+radiomic models (**Table 1**). The Gazi University Ethics Committee approved the study on April 16, 2024 (Research Code No: 2024-619).

**Table 1:** Machine learning model evaluations.

Models	Model AUC (Gradient Boosted Classification)	5-Fold Cross-Validation Mean AUC	AUC Standard Deviation (Cross-Validation)	Nonparametric Permutation Test p-value (Cross-Validation)
Clinical + Radiomic + Dosiomic	0.96	0.8	0.21	0.021
Clinical + Dosimetric	0.67	0.76	0.28	0.029
Clinical + Dosiomic	0.75	0.66	0.26	0.12
Clinical + Radiomic + Dosimetric	0.67	0.35	0.26	0.84
Clinical	0.89	0.38	0.33	0.77
Clinical + Radiomic	0.5	0.76	0.25	0.06

AUC, Area Under the Curv; p-value, Probability value from the t-test; indicating statistical significance (p ≤ 0,05).

### Study design

**Figure 2:**
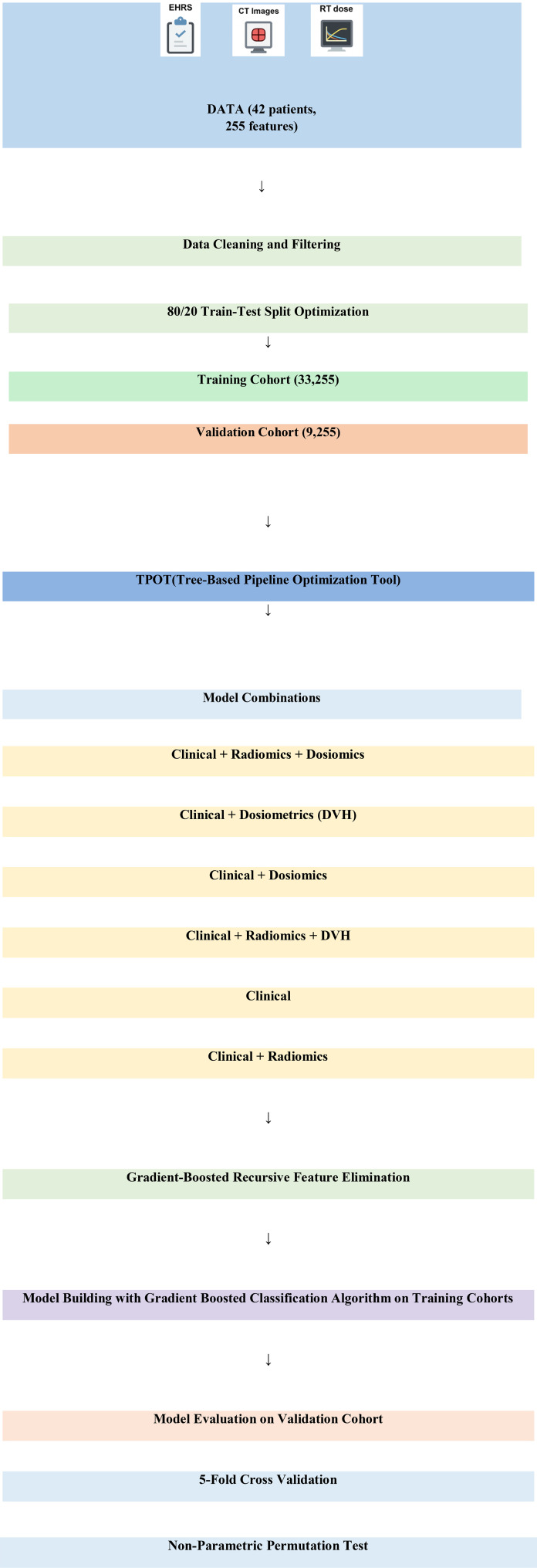
Overall workflow of the study. DVH, dose-volume histogram; EHRS, electronic hospital record system.

**Figure 2** illustrates the overall workflow of this study. Our study set the threshold value for high-sensitivity Troponin T (hs-TropT) at greater than 14 ng/L (23, 24). The dependent variable (target), hs-TropT, was classified into two groups: those above the threshold of 14 ng/L and those equal to or below 14 ng/L. Troponin-T levels exceeding 14 ng/dL were associated with treatment-related cardiac events. This proportion is consistent with the literature when considering our study data; hs-TropT values exceeding 14 ng/L comprised 16.66% of the patients (reported ranges in the literature vary between 10% and 47%) (15, 23). Sixteen dual (categorical) and five numerical variables were incorporated into the model, derived from dosimetric and clinical parameters. Clinical data were obtained from the electronic hospital record system, while dosimetric data were extracted from the dose-volume histograms (DVHs) of the 3D planned dose distributions. 119 radiomic and 111 dosiomic numerical features were individually extracted for each patient. Gradient-boosted recursive feature elimination, a hybrid method, was used to select important features for prediction.

### Clinical and dosimetric variables

The clinical data included patients’ age, comorbidities, smoking status, use of cardioprotective medications, administered chemotherapeutic agents, performed surgical procedures, and laterality (right or left breast). The dosimetric data encompassed the dose of radiotherapy delivered to the heart, the presence of a boost dose, fractionation scheme, radiotherapy technique employed, mean and maximum radiation doses received by the heart, and the heart volumes receiving 5 Gy and 25 Gy of radiation (V5 and V25) (2). Chemotherapy-related variables, including the administration of potentially cardiotoxic agents such as anthracyclines, trastuzumab, and taxanes, were incorporated into the model as binary categorical inputs (administered vs. not administered). Due to limitations in the available clinical data, cumulative dose information could not be retrieved and was therefore not used in the analysis.

### Medical image preprocessing and segmentation

3D dose distributions, radiotherapy planning CT scans, and organ contours were obtained from the Treatment Planning System (TPS). Before feature extraction, DICOM files from the TPS were converted to NRRD format using the plastimatch library (25). To address variations in slice thickness ranging from 2 mm to 5 mm, both dose distributions and CT images were resampled to an isotropic 1 mm resolution using B-spline interpolation. No filtering was applied; the original images were included in the model.

Voxel addition was performed for patients who underwent sequential radiotherapy to combine the boost dose with the whole-breast radiotherapy dose distributions using the SimpleITK library (26). During the extraction of these features, heart contours delineated by the patient’s physician in clinical practice were used (27). Dose and contouring files were processed in the plastimatch library for masking, and the ITK-SNAP interface was employed for accuracy control and visualization (28). Masking of CT images was conducted using the 3D Slicer interface (29). Patients with significant metal artifacts in their CT images were excluded from the study.

### Extraction of radiomic and dosiomic features

Radiomic features were extracted from 3D CT images using heart regions previously contoured by patients’ physicians during routine clinical practice, employing the 3D Slicer interface for segmentation, resampling, and feature extraction. Dose distributions were isotropically resampled to 1 mm within the Treatment Planning System, utilizing a cumulative dose intensity range of 1 Gy for dosiomic texture features and a voxel intensity bin width of 64 Hounsfield Units for radiomics. Features were extracted using the PyRadiomics library under eight categories, including First-Order Statistics, Shape Features, and various Gray Level Matrices (GLCM, GLDM, GLSZM, GLRLM, NGTDM), and are consistent with the Image Biomarker Standardisation Initiative (IBSI) guidelines (30).

### Model building and evaluation

Machine learning model selection and hyperparameter optimization were performed using the Tree-based Pipeline Optimization Tool (TPOT), an automated machine learning library in Python that utilizes genetic programming and evolutionary algorithms to identify optimal models and data processing pipelines (31, 32). The TPOTClassifier automatically explores a wide range of supervised classifiers including linear models (e.g., logistic regression), naïve Bayes variants, decision trees, ensemble methods (e.g., random forest, gradient boosting, XGBoost), and support vector machines. The model search is performed via genetic programming to select optimal preprocessing steps, feature selection, classifier type, and hyperparameters. Gradient-boosted classification yielded the highest validation AUC among the tested pipelines and was thus selected as the final model. Model performance was assessed using the area under the curve (AUC) method on data classified according to the hs-TropT> 14 ng/L threshold. To evaluate the model’s generalizability, we employed 5-fold cross-validation (33), and a non-parametric permutation test was conducted to assess the model’s robustness against randomness (34).

## Results

### Patient characteristics

42 patients (training: 33, validation: 9) were enrolled in this study. The characteristics of the patients are presented in **Table 2**. This was presented concerning hs-Troponin T levels, divided into four categories: those with hs-Troponin T levels above 14 ng/L and those below 14 ng/L, both with and without elevated clinical parameters.

**Table 2:** Clinical characteristics.

Clinical/Dosimetric Variable	Trop-T > 14 ng/L (+)	Trop-T ≤ 14 ng/L (+)	Trop-T > 14 ng/L (-)	Trop-T ≤ 14 ng/L (-)
Hypertension	3 (7%)	8 (19%)	4 (10%)	27 (64%)
Diabetes Mellitus	4 (10%)	6 (14%)	3 (7%)	29 (69%)
Smoking	1 (3%)	9 (21%)	3 (7%)	26 (62%)
Hyperlipidemia	1 (3%)	3 (7%)	6 (14%)	32 (76%)
ACE-i	0	1 (3%)	7 (17%)	34 (80%)
ARB	3 (7%)	6 (14%)	4 (10%)	29 (69%)
B-blocker	3 (7%)	3 (7%)	6 (14%)	30 (72%)
Trastuzumab	1 (3%)	6 (14%)	1 (3%)	34 (80%)
Cyclophosphamide	5 (12%)	24 (57%)	7 (17%)	11 (26%)
5FU/Capecitabine	0	0	3 (7%)	44 (93%)
Anthracycline	3 (7%)	27 (64%)	4 (10%)	24 (57%)
Taxane	5 (12%)	27 (64%)	4 (10%)	22 (52%)
Surgery (MRM)	5 (12%)	12 (28%)	4 (10%)	23 (55%)
RT Dose (2 Gy × 15)	4 (10%)	18 (43%)	8 (19%)	12 (28%)
Boost Dose (2 Gy × 5)	3 (7%)	12 (28%)	4 (10%)	23 (55%)
RT Technique (IMRT)	2 (5%)	19 (45%)	5 (12%)	16 (38%)

(+) and (-) indicate the presence and absence of the clinical condition or treatment, respectively; Trop-T, Troponin-T; ACE-i, Angiotensin Converting Enzyme Inhibitors; ARB, Angiotensin Receptor Blocker; MRM, Modified Radical Mastectomy; IMRT, Intensity Modulated Radiation Therapy.

### Dosiomic features and dose-volume factors

The features extracted from the original dose distributions in the treatment planning system through advanced statistical methods included first-order statistics (19), 2D shape features (16), 3D shape features (10), Gray Level Co-occurrence Matrix (GLCM) features (24), Gray Level Dependence Matrix (GLDM) features (16), Gray Level Size Zone Matrix (GLSZM) features (16), Gray Level Run Length Matrix (GLRLM) features (5), and Neighboring Gray Tone Difference Matrix (NGTDM) features (5), resulting in a total of 111 dosiomic based features calculated for each patient. The dosimetric features extracted from the heart contour are shown in **Table 3**.

**Table 3:** Dosimetric variables.

Dosimetric Features	Trop-T ≤ 14 (mean±std)	Trop-T > 14 (mean±std)	T-test p-value
Heart/Dmean	3.06±1.85	3.91±1.99	0.27
Heart/Dmax	31.26±17.76	30.76±13.96	0.94
Heart/V5	12.05±11.48	22.33±16.42	0.05
Heart/V25	2.53±3.21	1.28±1.85	0.32

Dmean, Mean dose; Dmax, Maximum dose; V5, Volume of the heart receiving 5 Gy or more; V25, Volume of the heart receiving 25 Gy or more; Trop-T, Troponin-T; mean±std, Mean value ± standard deviation; p-value, Probability value from the t-test, indicating statistical significance (p ≤ 0,05).

### Radiomic features

The features extracted from the CT scans taken for RT planning through advanced statistical methods included first-order statistics (18), 2D shape features (16), 3D shape features (10), Gray Level Co-occurrence Matrix (GLCM) features (24), Gray Level Dependence Matrix (GLDM) features (16), Gray Level Size Zone Matrix (GLSZM) features (16), Gray Level Run Length Matrix (GLRLM) features (5), and Neighboring Gray Tone Difference Matrix (NGTDM) features (14), resulting in a total of 119 radiomic features calculated for each patient.

### Univariate analysis

The chi-square test was used for univariate analysis of clinical-dosimetric categorical variables (**Table 2**). All variables were statistically non-significant for an association with elevated cardiac troponin (p>0.05). The independent samples t-test results indicated that the mean age was significantly higher in individuals with elevated troponin T levels (p = 0.044). Specifically, the mean age was 65.43 ± 14.57 years for those with Troponin T levels >14 ng/dL, compared to 55.31 ± 11.21 years for those with Troponin T levels ≤ 14 ng/dL. Additionally, an elevated heart V5 value was found to be borderline statistically significant in association with elevated troponin T levels among the dosimetric variables (p = 0,05) (**Table 3**). Recursive feature elimination was performed on six different models. The top five essential features extracted from the best-performing model (clinical + dosiomics + radiomics) were analyzed for their univariate association with troponin T elevation, as shown in **Table 4**.

**Table 4:** Most important features extracted from the most effective model (clinical + dosiomics + radiomics).

Biomarker	Classes	Features	T-test (Target = Troponin-T)
Dosiomic	First-order Statistics	Maximum	P<0.001
Radiomic	First-order Statistics	Minimum	P= 0.002
Dosiomic	First-order Statistics	10th Percentile	P= 0.406
Radiomic	Gray-Level Run Length Matrix	Run Entropy	P=0.159
Radiomic	First-order Statistics	Maximum	P=0.88

### Prediction performance

**Figure 3:**
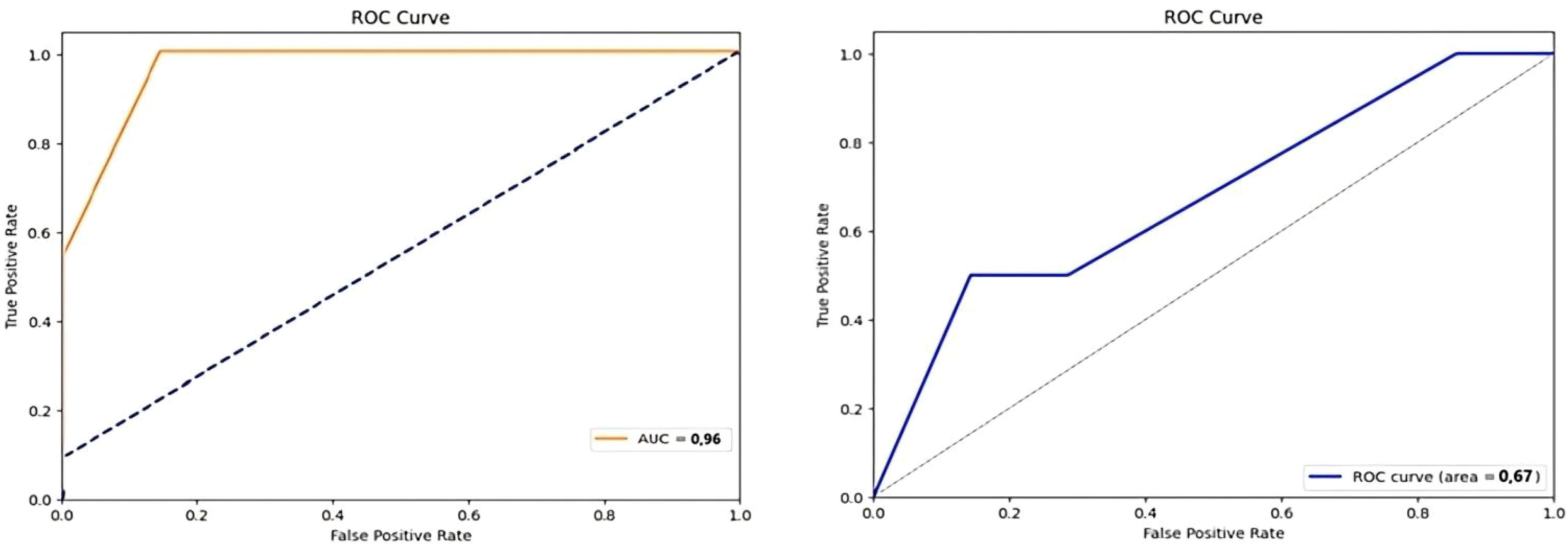
Receiver operating characteristic (ROC) curves for prediction models. Left: ROC curve for the clinical + radiomic + dosiomic model with an Area Under the Curve (AUC) of 0.96. Right: ROC curve for the clinical + dosimetric model with an AUC of 0.67. ROC, Receiver Operating Characteristic; AUC, Area Under the Carve.

The prediction models were evaluated using Gradient-Boosted Classification, with 5-fold cross-validation and non-parametric permutation tests to assess generalizability and model randomness. A total of six different models incorporating clinical, radiomic, dosiomic, and dosimetric parameters were constructed. The combined model of clinical, radiomic, and dosiomic parameters yielded the highest predictive performance, achieving an AUC of 0.96 for the validation cohort. This value was much lower for the clinical + dosimetric model (validation cohort-AUC = 0.67) (**Figure 3**). The internal validation using 5-fold cross-validation showed that the generalizability performance of the clinical + dosiomic + radiomic model was fair-to-good (mean AUC = 80.33 ± 21%). However, the relatively high standard deviation suggests considerable variability across folds, which may reflect model instability and potential overfitting due to the limited sample size. These two models were non-coincidences based on confirmation from nonparametric permutation tests (p<0,05). Other models were considered random based on permutation testing (p>0,05) (**Table 1**).

## Discussions

Traditional clinical studies in oncology typically rely on long-term follow-up and clinical, radiological, and dosimetric data. However, over the past decade, advancements in genomics and biomarker discovery have positioned personalized medicine as a central focus of scientific research. In 2017, the U.S. National Cancer Institute emphasized the potential of using biomarkers to evaluate the effects of radiotherapy on tissues, highlighting their capacity to revolutionize the assessment of treatment efficacy and average tissue damage (35). Despite the advances in precision medicine, predicting adverse events and disease prognosis using biomarkers remains an unmet need in medical research. This study focuses on predicting cardiac events that may arise as a result of breast cancer treatment before they become clinically evident. We utilized cardiac biomarkers, such as high-sensitivity Troponin T (hs-TropT), and imaging biomarkers derived from advanced statistical analyses of radiological images and dose distribution data (radiomics and dosiomics).

According to the literature, cardiovascular causes account for a significant proportion of deaths in breast cancer survivors (36, 37). Therefore, early prediction of cardiac events is essential for tailoring treatment and follow-up strategies in this patient population. While echocardiography remains the standard non-invasive method for detecting cancer therapy-related cardiac dysfunction (CTRCD) (7, 12), several studies suggest that it may not detect subclinical myocardial injury in its early stages, which can lead to missed opportunities for intervention (13, 14). The European Society of Cardiology has recommended that evaluating serum cardiac biomarkers, such as Troponin-T (Trop-T) and NT-proBNP, both at baseline and during or after treatment, may serve as a valuable diagnostic tool in the detection of subclinical cancer therapy-related cardiac dysfunction (CTRCD) and in tailoring oncological therapies based on individual risk profiles​ (16). Our findings corroborate previous reports, emphasizing the significance of imaging biomarkers like radiomics and dosiomics in improving early detection rates of such adverse events.

Our findings may emphasize the significant potential of models based on imaging biomarkers for early predicting cardiac events related to breast cancer treatment, compared to traditional clinical and dosimetric features. Furthermore, this may highlight the importance of a multidisciplinary approach, demonstrating that integrating clinical, dosimetric, imaging, and cardiological data can contribute to better management and personalization of patient treatment processes. The results of this study could provide a valuable foundation for future research focused on breast cancer treatment and side effect management.

Extracting three-dimensional (3D) radiomic and dosiomic features from imaging data provides a more comprehensive and objective assessment than traditional two-dimensional approaches. This study used PyRadiomics, an open-source Python library, to extract 3D features from medical images. While various methods can address calibration and noise issues in multi-center imaging studies, our images were acquired from a single center, minimizing these challenges. Consequently, we extracted radiomic features from the original planning CT images without additional filtering.

The only previous study utilizing dosiomics to investigate cardiac toxicity was conducted in a pediatric cohort to predict cardiac valvulopathy (38). This study is the first to develop a predictive model for adult breast cancer patients by integrating clinical, dosimetric, radiomic, and dosiomic data. The strength of this study is that no previous studies have specifically employed segmented cardiac dosiomic data in adult patients, underscoring the novelty of our research and its potential to serve as a foundation for future investigations.

Despite these promising results, our study has several limitations. The cumulative dose of cardiotoxic agents, particularly anthracyclines, is known to be strongly associated with cancer therapy-related cardiac dysfunction (CTRCD), with a recommended cumulative dose threshold of 550 mg/m². In our study, chemotherapy-related variables—including the use of anthracyclines, trastuzumab, and taxanes—were encoded as binary categorical inputs (administered vs. not administered). However, detailed cumulative dosing data were not available. This limitation may have hindered the predictive capacity of the clinical model regarding treatment-related cardiac toxicity (39, 40).

Moreover, the relatively small sample size, particularly the 9-patient validation cohort, presents a limitation regarding model generalizability and the potential risk of overfitting. As this study was conducted within the scope of a completed and ethically approved academic thesis, adding new patients retrospectively or implementing synthetic data augmentation techniques (e.g., SMOTE) was not feasible. Therefore, while the findings are promising, they should be interpreted as exploratory and hypothesis-generating, warranting validation in larger prospective cohorts.

This limitation was also reflected in the internal validation results. The relatively high standard deviation in AUC values across the cross-validation folds (80.33 ± 21%) indicates potential model instability and overfitting risk. Such variability is commonly observed in small datasets, and highlights the importance of future validation using larger, independent cohorts or more robust validation schemes such as repeated or nested cross-validation. Future studies should employ repeated or nested cross-validation frameworks to enhance the reliability of performance estimates, especially in datasets with limited sample sizes.

Small-sample machine learning studies are increasingly reported in emerging fields such as cardio-oncology, where access to large annotated datasets is limited. Despite their limitations, such studies often play a critical role in hypothesis generation and feasibility assessment, especially when novel biomarkers or data types are explored. In our case, the integration of heart-segmented dosiomics represents a unique and innovative dimension that justifies the use of a pilot-scale dataset. We believe that our findings contribute valuable preliminary insights that warrant future validation in larger, prospective studies.

However, dosiomic features were extracted from the original treatment plan’s dose distribution, and the model still achieved high performance (AUC = 0.96). Extracting features from Biological Effective Dose (BED) distributions could be considered as an alternative approach. Evaluating the impact of BED-based dosiomic features on model performance warrants further investigation in future studies.

Class imbalance is a significant challenge in medical side-effect prediction studies involving machine learning. In our dataset, the target variable hs-TropT> 14 ng/L represented only 16.66% of the data, resulting in an imbalanced dataset. Class imbalance can cause models to favor the majority class, thereby reducing accuracy in predicting minority class instances. Gradient-boosted classification algorithms help mitigate class imbalance by automatically adjusting weights to define minority classes better, and the AUC metric remains unaffected by class imbalance. However, despite using these methods, performance in other models remained lower, suggesting that alternative techniques (e.g., synthetic data generation or data reduction) may be necessary to address the imbalance, representing a key area for improvement in our study.

Another challenge in radiomic studies is respiratory and cardiac motion, which can reduce the quality of extracted features. Our study did not include patients with controlled breathing; future research could explore this area. Moreover, considering the potential of respiratory control to reduce cardiac events, evaluating its impact on predictive models would be valuable. We also excluded patients with significant metal artifacts in their planning CT scans, as such artifacts could affect both dose distribution and radiomic feature extraction. Investigating prediction models for this patient group remains an area for future research.

## Future perspectives

This study highlights the potential of integrating imaging biomarkers such as radiomic and dosiomic features into predictive models for early detection of treatment-related cardiac events in breast cancer patients. However, several areas require further investigation to realize these models’ clinical applicability fully. One key area for future research is the inclusion of more precise cumulative anthracycline doses in predictive models. As anthracycline dosage is closely associated with cancer therapy-related cardiac dysfunction (CTRCD), moving beyond categorical variables to incorporate exact dosage values could significantly enhance the predictive accuracy of such models. This will enable more personalized treatment adjustments, potentially mitigating the risk of cardiac events for high-risk patients.

Another avenue for future research is the exploration of Biological Effective Dose (BED) distributions for dosiomic feature extraction. While our study demonstrated high model performance using traditional dose distributions, BED-based dosiomics may provide an even more accurate representation of the actual biological impact of radiotherapy on cardiac tissues. Evaluating the contribution of BED-based dosiomic features could improve model precision and further tailor treatment plans to individual patient risk profiles.

Moreover, future studies should aim to conduct more extensive, multi-center investigations to validate the generalizability of the proposed models across diverse patient populations and clinical settings. This broader scope would provide more robust evidence and ensure that the models are applicable in varied healthcare environments. Additionally, incorporating the effects of cardiac and respiratory motion into predictive models is essential. Since these motions can impact dose distribution and radiomic feature extraction, addressing these factors in future research could significantly enhance model accuracy and the early detection of subclinical cardiac events.

Lastly, addressing the challenge of class imbalance remains critical in enhancing the robustness of machine learning models in medical side-effect studies. Future studies should explore alternative techniques, such as synthetic data generation or advanced data reduction methods, to mitigate this issue. By overcoming these challenges and continuing to refine predictive models by integrating diverse clinical, imaging, and dosimetric data, future research could significantly improve the prediction and prevention of treatment-related cardiac events, ultimately leading to better patient outcomes and personalized medicine applications.

## Conclusions

This study can demonstrate that imaging biomarkers (radiomic and dosiomic parameters) significantly outperform traditional clinical and dosimetric parameters in predicting treatment-related cardiac events in breast cancer patients (96% vs. 67% AUC). When integrated into clinical practice, these radiomic and dosiomic parameters could become critical patient factors, potentially altering treatment and follow-up strategies by predicting cardiac side effects with nearly perfect (%96) accuracy. However, the moderate-to-good generalizability observed in cross-validation suggests that these models need to be supported by real-world data. This underscores the necessity for further research to integrate imaging biomarkers, which currently require complex statistical processes and have yet to be widely adopted in personalized medicine applications.

## Data Availability

The original contributions presented in the study are included in the article. Further inquiries can be directed to the corresponding author.

## Glossary

ACE-i: Angiotensin Converting Enzyme Inhibitors
ARB: Angiotensin Receptor Blocker
AUC: Area Under the Curve
BED: Biological Effective Dose
CTRCD: Cancer Therapy-Related Cardiac Dysfunction
CT: Computed Tomography
DICOM: Digital Imaging and Communications in Medicine
DVH: Dose-Volume Histogram
Dmax: Maximum Dose;
Dmean: Mean Dose
EHRS: Electronic Hospital Record System
GLCM: Gray Level Co-occurrence Matrix
GLDM: Gray Level Dependence Matrix
GLRLM: Gray Level Run Length Matrix
GLSZM: Gray Level Size Zone Matrix
hs-TropT: High-Sensitivity Troponin-T
IBSI: Image Biomarker Standardization Initiative
IMRT: Intensity Modulated Radiation Therapy
ITK: Insight Segmentation and Registration Toolkit
LVEF: Left Ventricular Ejection Fraction
MRM: Modified Radical Mastectomy
NGTDM: Neighboring Gray Tone Difference Matrix
NT-proBNP: N-Terminal Pro B-Type Natriuretic Peptide
PET: Positron Emission Tomography
PyRadiomics: Python-based Radiomics Extraction Library
ROC: Receiver Operating Characteristic
RT: Radiotherapy
TPS: Treatment Planning System
TPOT: Tree-Based Pipeline Optimization Tool
V5: Volume of the Heart Receiving 5 Gy or More
V25: Volume of the Heart Receiving 25 Gy or More
